# Volumetric printing and non-destructive drug quantification of water-soluble supramolecular hydrogels

**DOI:** 10.1007/s13346-024-01723-6

**Published:** 2024-10-18

**Authors:** Jun Jie Ong, Anna Kirstine Jørgensen, Zilan Zhu, Richard Telford, Philip J. Davies, Simon Gaisford, Alvaro Goyanes, Abdul W. Basit

**Affiliations:** 1 Department of Pharmaceutics, UCL School of Pharmacy, University College London, 29-39 Brunswick Square, London, WC1N 1AX UK; 2 Departamento de Farmacología, Farmacia y Tecnología Farmacéutica, Facultad de Farmacia, Instituto de Materiales (iMATUS) and Health Research Institute of Santiago de Compostela (IDIS), Universidade de Santiago de Compostela, Santiago de Compostela, 15782 Spain; 3 School of Chemistry and Biosciences, University of Bradford, Richmond Road, Bradford, BD7 1DP UK; 4 TA Instruments, a Division of Waters Ltd, Stamford Avenue, Altrincham Road, Wilmslow, Cheshire, SK9 4AX UK; 5 FABRX Ltd, Henwood House, Henwood, Ashford, TN24 8DH UK

**Keywords:** Vat photopolymerization additive manufacturing, Personalized oral drug products, Three dimensional printed pharmaceuticals and medications, Process analytical technology and quality control, Clinical translation of printed drug delivery systems and medicines

## Abstract

Vat photopolymerisation 3D printing is being actively explored for manufacturing personalised medicines due to its high dimensional accuracy and lack of heat application. However, several challenges have hindered its clinical translation, including the inadequate printing speeds, the lack of resins that give soluble matrices, and the need for non-destructive quality control measures. In this study, for the first time, a rapid approach to producing water-soluble vat photopolymerised matrices and a means of non-destructively verifying their drug content were investigated. Volumetric printing, a novel form of vat photopolymerisation, was used to fabricate personalised warfarin-loaded 3D-printed tablets (printlets). Eight different formulations containing varying amounts of warfarin (0.5–6.0% w/w) were used to print two different sized torus-shaped printlets within 6.5 to 11.1 s. Nuclear magnetic resonance (NMR) spectroscopy revealed the presence of only trace amounts of unreacted acrylate monomers, suggesting that the photopolymerisation reaction had occurred to near completion. All printlets completely solubilised and released their entire drug load within 2.5 to 7 h. NIR spectroscopy (NIRS) was used to non-destructively verify the dose of warfarin loaded into the vat photopolymerised printlets. The partial least square regression model built showed strong linearity (R^2^ = 0.980), and high accuracy in predicting the drug loading of the test sample (RMSEP = 0.205%). Therefore, this study advances pharmaceutical vat photopolymerisation by demonstrating the feasibility of producing water-soluble printlets via volumetric printing and quantifying the drug load of vat photopolymerised printlets with NIRS.

**Graphical abstract:**

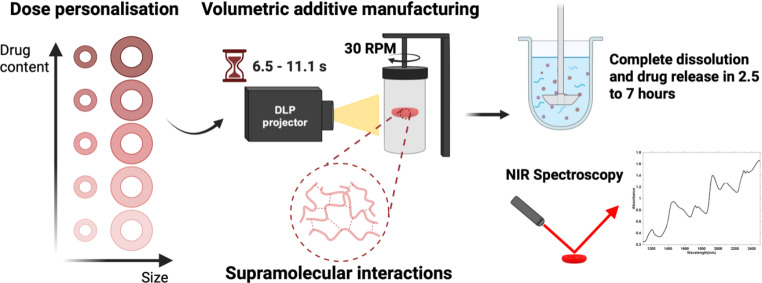

## Introduction

Personalised medicine is a modern practice in healthcare where medical care and interventions are tailored to individual patients as opposed to a “one-size-fits-all” approach based on population means [1, 2]. While personalised medicines emerged in the past two decades following notable advancements in molecular genetics, personalising a patient’s drug treatments has been and remains in practice since the 1960s in the form of therapeutic drug monitoring (TDM) [3–5]. TDM is the clinical practice of adjusting a patient’s drug regime based on their serum, plasma, or whole blood drug concentration [5]. This is primarily applied to drugs that have small differences in their therapeutic and toxic dose, i.e., narrow therapeutic index (NTI) drugs. For example, warfarin is an NTI anti-coagulant drug where individual dose requirements can vary significantly, based on the patient’s age, body mass index, and genetic variations in enzymes (cytochrome P450) that metabolise the drug [6]. Consequently, a patient’s prescribed dose of warfarin is based on their international normalised ratio (INR), which indicates the time it takes for blood to clot. With growing pharmacogenomic evidence and emerging healthcare technologies, the practice of TDM and medicines personalisation is expected to see improvements in dose optimisation, therapeutic outcomes, and accessibility.

3D printing (3DP), or additive manufacturing, is one such technology that is being actively explored in the pharmaceutical field for its ability to fabricate personalised medical devices and medicines [7–11]. Specific to the latter, the technology allows medicines to be tailored in terms of dose, drug release profile, and geometry according to the patient’s therapeutic needs. Amongst the various categories of 3D printing, vat photopolymerisation affords high spatial resolution without necessitating the application of heat [12–14]. Vat photopolymerisation-based technologies, such as stereolithography (SLA), digital light processing (DLP) and liquid crystal display (LCD), use light to induce photopolymerisation and hence solidification of a liquid photosensitive resin [15, 16]. Owing to its high dimensional accuracy and compatibility with heat labile drugs, this technology has been explored for numerous pharmaceutical and healthcare applications, such as dental implants [17–19], personalised tablets (printlets) [20–23], patient-tailored drug-eluting devices [24–26], and microneedles [27–29]. However, due to the layer-by-layer means of fabrication, SLA, DLP, and LCD 3DP may not be able to provide the printing speeds required to keep up and be deployed in fast-paced clinical settings.

Volumetric printing, or volumetric additive manufacturing, is a novel type of vat photopolymerisation printing that affords significantly faster printing speeds [30–32]. Unlike SLA and DLP, volumetric printing does not produce the desired 3D geometry layer-by-layer; instead, the entire object is fabricated simultaneously through the accumulation of light patterns derived from images of the object viewed from different angles. In the pharmaceutical space, volumetric printing has been used to fabricate paracetamol-loaded tablets in as little as 7 s, which is significantly faster than DLP and SLA that requires the same time to polymerise a single layer [33–35]. However, as with other vat photopolymerisation printing technologies, drug delivery devices and dosage forms fabricated in this way have been insoluble in water. Consequently, applications have been limited to sustained release medicines and drug-eluting medical devices that do not require solubilisation of the printed matrix. Recently, our group reported a novel vat photopolymerisable formulation that produced water-soluble paracetamol-loaded matrices printed through DLP 3DP [36]. Preliminary evidence from a single-dose acute toxicity animal study on matrices derived from this formulation also provided early evidence on the safety of the photopolymerised polymers. However, as volumetric printing has resin requirements that are distinct from DLP and SLA printing (e.g., resin transparency), the feasibility of printing matrices with this novel formulation with volumetric printing remains to be investigated.

Another barrier to the clinical translation of vat photopolymerisation 3DP, and other pharmaceutical 3DP technologies, is the challenge of safeguarding and validating the quality of made-to-order medicines [37]. As these personalised medicines are not made in excess, conventional quality control measures that are destructive, such as drug quantification via high performance liquid chromatography or UV spectroscopy assays, are not suitable. Near infra-red spectroscopy (NIRS) is an analytical technology that has been explored for non-destructive dose verification of 3D printed medicines [38, 39]. Every compound has a unique NIR spectrum, where the intrinsic peaks of the compound can be correlated to its concentration through multivariate modelling. Thus, quantitative multivariate models can be built to determine the percentage content of a drug within a 3D printed matrix. This has successfully been used for drug quantification in selective laser sintering printed tablets [40, 41], inkjet-printed devices [42], and direct powder extrusion printed medicines [43]. A drawback of NIRS is that the analyte signals can be easily overwhelmed by the strong overtone and combination bands of vibrations arising from water molecules, as water strongly absorbs energy in the IR region [44, 45]. Consequently, quantifying the drug load of vat photopolymerised hydrogels (herein referred to as printlets) with NIRS could be a challenge given the common inclusion of water as a non-reactive diluent.

Therefore, this study aims to investigate the feasibility of using volumetric printing to fabricate personalised warfarin sodium-loaded water-soluble printlets via volumetric printing using the novel water-soluble matrix formulation. This study represents the first-time water-soluble warfarin-loaded printlets have been fabricated using vat photopolymerization. The amount of warfarin sodium loaded into the resin mixture and the size of the printlets were varied to demonstrate the potential to personalise printlets according to the patient’s INR. NIRS was used to non-destructively quantify the weight% of warfarin sodium loaded into the printlets, representing the first time NIRS is used for dose verification of vat photopolymerised printlets.

## Materials and methods

### Materials

[2-(Acryloyloxy)ethyl]trimethylammonium chloride solution (TMAEA) (80 wt% in water), lithium phenyl-2,4,6-trimethylbenzoylphosphinate (LAP, MW 294.21 g/mol, ≥ 95%), 2-propanol (isopropanol, puriss, ≥ 99.8%), acetonitrile (ACN) for HPLC (gradient grade, ≥ 99.9%), and deuterium oxide (99.9 atom % D) were purchased from Sigma-Aldrich (Dorset, UK). Warfarin sodium clathrate (MW 330.31 g/mol, > 98.0%) was purchased from LKT Laboratories Inc. (St. Paul MN, USA). Red food colorant (*Kroma Kolors*, Kopykake, Torrance, CA, USA) was purchased from Shesto Limited (Watford, UK). Sodium acetate (MW 82.03 g/mol) was purchased from VWR Chemicals (Leuven, Belgium). Glacial acetic acid (MW 60.05 g/mol) was purchased from Severn Biotech Ltd. (Worcestershire, UK). Hydrochloric acid 1 M solution was purchased from LP Chemicals Ltd (Winsford, UK). All materials were used as received.

### Preparation of formulation

Eight different formulations with different concentrations of warfarin sodium were prepared (Table 1). 80 g of resin was prepared for each of the formulations using an analytical balance. LAP, red food colourant, and warfarin sodium were dissolved in water in an amber glass bottle before the addition of TMAEA. The resins were left to stir with a magnetic stirrer bar at room temperature overnight (~ 12 h), and were used for printing immediately after preparation.

**Table 1:** Compositions of the formulations. The numbers used in the formulation codes represent the concentration of warfarin sodium that were included, i.e. VOL005 contains 0.5% w/w of warfarin sodium

Formulation	TMAEA (% w/w)	Warfarin sodium (% w/w)	Water (% w/w)	LAP (% w/w)	Food colorant (% w/w)
VOL005	93.45	0.5	6	0.025	0.025
VOL010	92.95	1.0	6	0.025	0.025
VOL020	91.95	2.0	6	0.025	0.025
VOL030	90.95	3.0	6	0.025	0.025
VOL035*	90.45	3.5	6	0.025	0.025
VOL040	89.95	4.0	6	0.025	0.025
VOL050	88.95	5.0	6	0.025	0.025
VOL060	87.95	6.0	6	0.025	0.025

* Indicates formulations used for external validation of NIR calibration model

### Optical density measurement

The optical density of the resin formulations and their equivalent without 6% w/w water were measured to investigate the impact of pre-solubilising warfarin sodium before addition of TMAEA. Approximately 1 mL of resin was transferred into 4-Clear Faces Macro Cuvettes (Fisher Scientific, UK) and their optical density at wavelength 600 nm (OD_600_) were measured using a Cary 100 UV/Vis spectrophotometer (Agilent Technologies, UK). Resins were analysed immediately after preparation and all measurements were performed in triplicates.

### Volumetric 3D printing

**Fig. 1:**
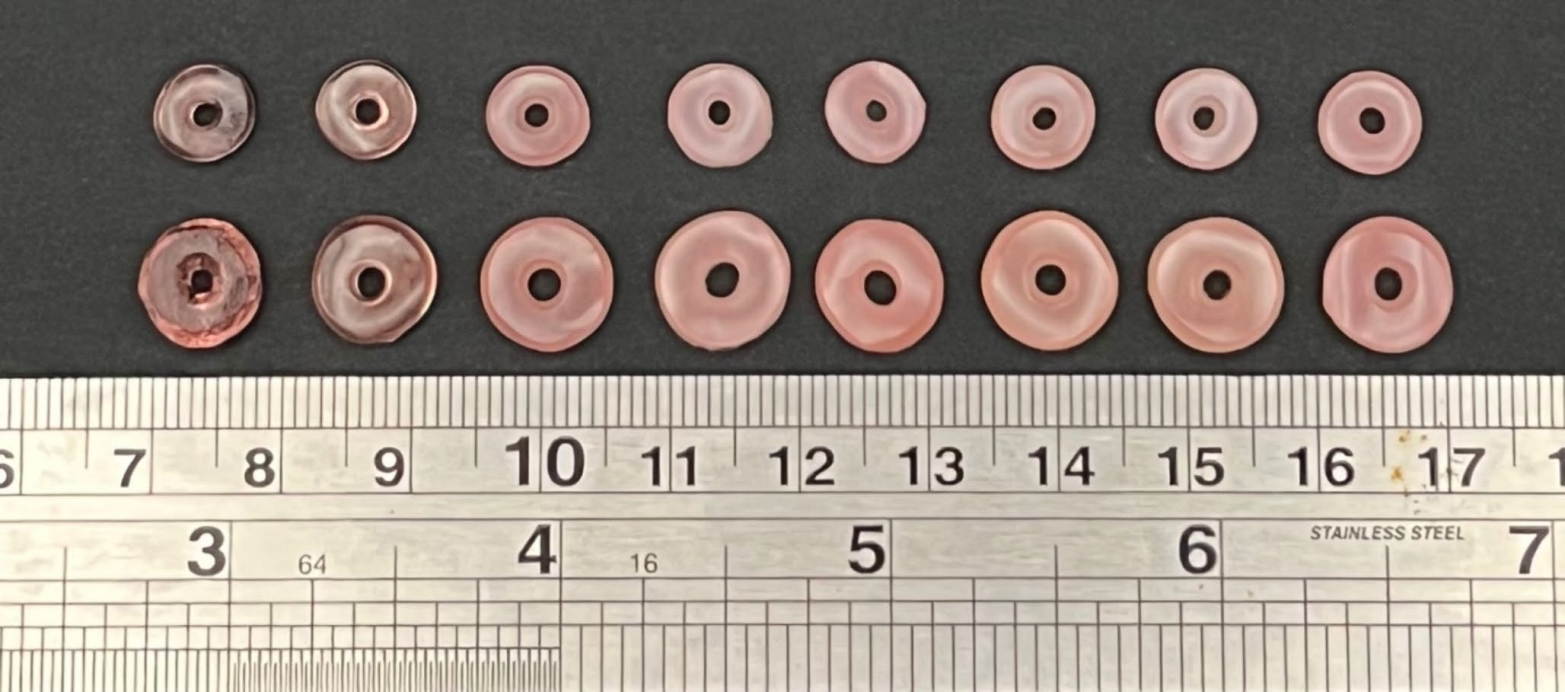
Pictures of (from left to right) VOL005, VOL010, VOL020, VOL030, VOL035, VOL040, VOL050, and VOL060 printlets. Small-sized printlets are positioned on the top row, while large-sized printlets are positioned on the bottom row. Scale in cm

The volumetric printer (FabRx Ltd., United Kingdom) consisted of a digital light projector (DLP) (Wintech DLP6500, USA), emitting 385 nm UV light in the direction of the rotating cylindrical resin container (2.5 cm diameter x 5 cm height) (Fig. 1) which was suspended 23 cm from the DLP and 15 cm from the base by an axis attached to a motor allowing 360° rotation.

Two different sized torus shapes, a large (12.8 mm diameter x 5.6 mm height) and a small (10.24 mm diameter x 4.48 mm height), were printed for each formulation. The small torus was 20% smaller in dimensions than the large torus, approximately halving its weight and volume (volume of torus = 2π^2^Rr^2^, where R is the major radius and r is the minor radius). Two identical circles were created using Microsoft Paint (Version 6.3, build 9600), and loaded into a software designed by FabRx (London, UK) that controls the printer and projects the two-dimensional image onto the resin container, resulting in the desired 3D structure.

The photosensitive resin (as prepared in the Preparation of formulation section) was introduced into the container, which was then attached to the rotary motor via the axis support. The exposure time, rotation speed, and brightness used to print each formulation is summarised in Table 2. After printing, printlets were rinsed for 1 min in isopropyl alcohol (IPA), and post-cured in a Form Cure (Formlabs Inc., Somerville, MA, USA) at room temperature (~ 25 ºC) for 30 min.

**Table 2:** Printing settings for each formulation

Formulation	Torus size	Rotation speed (RPM)	Brightness (lx)	Exposure time (s)
VOL005	Large	30	230*	6.5
	Small	30	230	6.5
VOL010	Large	30	230	6.8
	Small	30	230	6.8
VOL020	Large	30	230	8.0
	Small	30	230	8.0
VOL030	Large	30	230	8.6
	Small	30	230	8.6
VOL035	Large	30	230	9.3
	Small	30	230	9.3
VOL040	Large	30	230	9.6
	Small	30	230	9.6
VOL050	Large	30	230	9.9
	Small	30	230	9.9
VOL060	Large	30	230	11.1
	Small	30	230	11.1

*Equivalent to 62.5% of Wintech DLP6500 maximum brightness

### Drug loading

#### In printlet

Printlets were dissolved in distilled water in 100 mL volumetric flasks and placed under magnetic stirring for 24 h (*n* = 3). 1 mL of sample was withdrawn and centrifuged at 15,000 xg for 5 min. The supernatant was diluted 10x with distilled water for quantification by HPLC, as described in the High-performance liquid chromatography (HPLC) section.

#### In photosensitive resins

Approximately 0.05 g of each photosensitive resin was accurately weighed out and dissolved in distilled water in 100 mL volumetric flasks, which were left under magnetic stirring for 24 h (*n* = 3). 1 mL of samples were subsequently withdrawn and centrifuged at 15,000 xg for 5 min. The supernatant was analysed via HPLC (see High-performance liquid chromatography (HPLC) section).

### Near infra-red spectroscopy

NIR reflectance spectra were acquired using a MicroNIR 1700ES NIR spectrometer (VIAVI, Hertfordshire, UK) equipped with 2 vacuum tungsten lamps and an InGaAs photodiode array detector between 908 − 1,676 nm (11,012 − 5,966 cm^− 1^) using a probe with an 8 mm diameter collection optic. Printlets were placed on fine structured crepe paper tape coated with a UV resistant acrylic adhesive (*Manutan*, Dorset, UK), and the probe was positioned on top of the printlet. 10 NIR reflectance spectra were obtained for each printlet. The fine structured crepe tape was used for the acquisition of dark and reference spectra for instrument calibration.

The raw spectra were pre-processed by detrend scatter correction with a breakpoint at the sixth spectral point and taking the first derivative spectra by application of Savitzky-Golay filter (window size of seven, second order polynomial). Partial least squares regression (PLSR) was performed using eight latent variables, with 10-fold cross validation, to build the calibration model.

### X-ray powder diffraction (XRPD)

XRPD was performed to investigate the solid state of warfarin in the printlets. Discs (9.0 mm diameter x 1.0 mm height) were 3D-printed using each resin formulation with the same printing settings as those used to prepare the respective printlets to assess the solid state of warfarin in the printlets. Printed discs and pure warfarin sodium powder were analysed by XRPD with a Rigaku MiniFlex 600 (Rigaku, Wilmington, MA, USA) equipped with a Cu X199 ray source (λ = 1.5418 Å). The intensity and voltage applied were 15 mA and 40 kV. Samples were scanned between 2θ = 3–60° with a stepwise size of 0.02° at a speed of 5°/min.

### Thermal analysis

Differential scanning calorimetry (DSC) measurements were performed with a Q2000 DSC (TA instruments, Waters, LLC, New Castle, DE, USA) to characterise warfarin sodium powder and printlets. Nitrogen was used as a purge gas with a flow rate of 50.0 mL/min. The samples were heated from 0 to 225 °C, then cooled to 0 ºC, and then reheated to 225 ºC all at a rate of 10 ºC/min. Data were collected with TA Advantage software for Q series (version 2.8.394, TA instruments, Waters LLC, New Castle, DE, USA) and analysed using TA Instruments Universal Analysis 2000. TA aluminium pans and pin-holed hermetic lids (Tzero) were used with an average sample size of 3.0–5.0 mg.

Thermogravimetric analysis (TGA) was performed with a Discovery TGA (TA Instruments, Waters LLC, USA). Samples were heated from 30 to 225 ºC at 10 ºC/min in open aluminium pans, and nitrogen was used as a purge gas with a flow rate of 25 mL/min. TGA-MS experiments were performed on a Discovery TGA5500 (TA Instruments, Waters LLC, USA) hyphenated to a Pfeiffer Vacuum Thermostar GSD350 mass spectrometer (Pfeiffer Vacuum GmbH, Germany) through a heated capillary held at 200 °C. Samples (ca. 20 mg) were held isothermally at room temperature for 5 min before heating to 250 °C at 10 °C/min under a helium purge gas with a flow rate of 25 mL/min. 100 µL platinum pans were used. A scanning mass spectrometry method was used to record data between m/z 5 and 100 (64 ms dwell time at each mass). A second multiple ion detection scan (MID) method was used for a repeat of the warfarin sodium sample to improve resolution (same TGA conditions) monitoring ions at m/z 18 (water) and 27, 29, 43 and 45 (isopropylalcohol) with a dwell time of 256 ms at each mass. Data collection and analysis were performed using TA Instruments Trios software.

### Fourier-transform infrared spectroscopy (FTIR)

The infrared spectra of formulations before and after volumetric 3D printing were collected over a range of 4000–650 cm^− 1^ at a resolution of 4 cm^− 1^ for 6 scans using a Spectrum 100 FTIR spectrometer (PerkinElmer, Waltham, MA, USA). The spectra of pure warfarin sodium powder, LAP powder, food colourant, and TMAEA were collected as references.

### Nuclear Magnetic Resonance (NMR) spectroscopy

All NMR spectra were recorded in 99.9% D2O. ^1^H-NMR spectra of TMAEA, LAP, warfarin sodium, food colourant, and printlet samples were obtained separately. 10 mg of sample was dissolved in 1 mL of D_2_O for analysis. ^1^H-NMR spectra of the samples were obtained using a Bruker AVANCE 400 spectrometer (Bruker, UK). Data acquisition and processing were performed using standard TopSpin Software (Bruker, UK).

### In vitro drug release

Dissolution profiles for each type of printlets were obtained using USP-II apparatus (Model PTWS, Pharmatest, Germany) (*n* = 3) set at 50 rpm and 37 ± 0.5 °C. For the first 2 h, samples were placed in 750 mL of 0.1 M HCl. After 2 h, 250 mL of 0.2 M trisodium phosphate solution was added to each dissolution vessel and the pH was adjusted to 6.8 using 5.0 M NaOH solution. 1.0 mL samples were withdrawn at 5, 10, 20, 30, 45, 60, 90, 120, 150, 180, 240, 300, 360, 420, and 480 min and centrifuged at 15,000 xg for 5 min. The supernatant was analysed using HPLC, as described in the High-performance liquid chromatography (HPLC) section.

### High-Performance Liquid Chromatography (HPLC)

A Hewlett Packard 1260 Series HPLC system equipped with an online degasser, quaternary pump, column heater, autosampler and UV/Vis detector, was used. 50 µL of the samples were injected into an Eclipse plus C18 5 μm column, 4.6 × 150 mm (Zorbax, Agilent technologies, Cheshire, UK) at 30 °C. The mobile phase consisted of 50 mM acetate buffer (pH 5.5) and ACN at a flow rate of 1 mL/ min under the gradient program as follows: 15% (v/v) ACN increased to 60% (v/v) in 5 min and decreased to 15% (v/v) in 1 min and held for 4 min prior to the next injection. The total run time was 10 min, eluents were detected at 300 nm.

### Statistical analysis

Data were reported throughout as mean ± standard deviation (*n* = 3, unless otherwise stated). Two sample t-test at 95% significance level (*p* < 0.05 respectively) was used to analyse statistically significant difference between sample groups.

## Results and discussion

In tomographic volumetric 3DP, images of the desired 3D object viewed from different angles are projected in synchronisation with the rotation of the resin container. For the projected light patterns to be distributed at the intended location, the photosensitive resin must be transparent or considerable corrections to the projected images are needed to account for the expected distortion [31, 32]. The presence of particles or micelles can cause light to scatter, resulting in distorted light patterns and inaccurate prints.

Formulations were initially prepared without water; however, due to warfarin insolubility in TMAEA, these were turbid and resulted in significant light scattering (OD_600_ = 1.91 ± 0.0113 abs) that prevented volumetric printing of consistent and well-formed shapes. Pre-solubilisation of warfarin sodium load in distilled water prior to the addition of TMAEA yielded transparent resins (OD_600_ = 0.00131 ± 0.00731 abs), significantly lower (*p* < 0.0001) based on two sample t-test than the resin without water, and suitable for volumetric printing [46]. Water addition was kept minimal in the formulations in this study: 6% w/w water would yield a saturated solution of warfarin sodium (1 mg/mL) for the formulation with the highest expected drug load (VOL060) [47]. Our previous study found that the inclusion of water impaired the structural integrity of supramolecular printlets derived from TMAEA, due to the disruption of inter-chain supramolecular interactions by water molecules, and thus the relative proportion of water was kept constant in all formulations to avoid impacting drug release and physical properties of the printlets [36].

Volumetric printing of the optimised formulations successfully produced torus-shaped printlets of two different sizes, with consistent dimensions and weight (for large printlets: 3.60 ± 0.064 mm in height, 9.43 ± 0.231 in diameter, 233.88 ± 13.33 mg; for small printlets: 2.94 ± 0.083 mm in height, 7.28 ± 0.14 mm in diameter, 113.97 ± 6.34 mg) (Fig. 1; Table 3). The slight variation in dimensional precision for the printing method is likely a consequence of the water in the resin formulation, resulting in less supramolecular inter-polymer interactions and thereby less densely packed printlets [36]. The printlets were slightly smaller than the projected images, as the convex surface of the resin container resulted in variable light refraction depending on the point at which light strikes the container surface, resulting in a smaller than programmed light pattern delivered inside container [33]. The exposure time required to fabricate the printlets increased with the relative proportion of warfarin sodium in the formulation, ranging from 6.5 s for VOL005 to 11.1 s for VOL060 (Table 2). This may be attributed to warfarin sodium being another non-reactive component in the resin mixture besides the included water [48]. As such, as drug loading increases, the concentration of TMAEA decreases, causing the rate of photopolymerisation to decrease and necessitating a longer exposure time to achieve the same degree of curing.

The combination of eight different drug loadings and two sizes allowed the fabrication of warfarin sodium printlets ranging from 0.462 to 14.4 mg in drug content, covering the range of warfarin sodium doses that are commercially available as tablets (0.5–10 mg). Therefore, the warfarin sodium printlets reported in this study may be personalised according to a patient’s international normalised ratio (INR) of prothrombin time. The dimensions of smaller printlet were chosen such that its volume would be half that of the larger printlet, in line with the common practice of breaking commercial warfarin tablets in half to adjust the dose. The choice of two substantially different sized torus also permitted investigation on the effect of surface area to volume ratio on the printlets’ drug release rates.

**Table 3:** Physical dimensions and weight of printlets, resin and printlet drug loading determined via HPLC

Formulation	Diameter (mm) (*n =* 8)	Height (mm) (*n =* 8)	Weight (mg) (*n =* 8)	Resin loading (% w/w) (*n =* 3)	Printlet loading (% w/w) (*n =* 3)
VOL005L	9.65 ± 0.14	3.64 ± 0.05	239.00 ± 5.65	0.485 ± 0.00335	0.456 ± 0.0105
VOL005S	7.25 ± 0.11	2.90 ± 0.10	110.83 ± 2.04		0.417 ± 0.0116
VOL010L	9.23 ± 0.17	3.58 ± 0.05	222.06 ± 6.93	0.970 ± 0.00785	0.942 ± 0.0499
VOL010S	7.18 ± 0.13	2.93 ± 0.07	111.91 ± 1.41		0.883 ± 0.0221
VOL020L	9.63 ± 0.10	3.68 ± 0.09	245.12 ± 1.65	1.95 ± 0.000319	2.01 ± 0.0308
VOL020S	7.30 ± 0.11	2.99 ± 0.06	121.34 ± 1.85		1.92 ± 0.0450
VOL030L	9.12 ± 0.13	3.58 ± 0.07	224.51 ± 4.72	2.92 ± 0.0319	3.05 ± 0.0133
VOL030S	7.29 ± 0.14	2.9 ± 0.08	112.1 ± 1.75		2.85 ± 0.0726
VOL035L	9.46 ± 0.18	3.58 ± 0.05	230.71 ± 7.29	3.43 ± 0.00763	3.71 ± 0.00962
VOL035S	7.21 ± 0.20	2.96 ± 0.05	109.1 ± 4.48		3.46 ± 0.0775
VOL040L	9.70 ± 0.05	3.59 ± 0.04	258.05 ± 2.89	3.87 ± 0.0348	4.19 ± 0.0688
VOL040S	7.36 ± 0.07	3.04 ± 0.05	125.3 ± 1.32		3.95 ± 0.0908
VOL050L	9.26 ± 0.17	3.58 ± 0.05	221.40 ± 7.47	4.90 ± 0.0462	5.24 ± 0.123
VOL050S	7.24 ± 0.17	2.86 ± 0.05	107.9 ± 4.18		4.83 ± 0.260
VOL060L	9.48 ± 0.09	3.60 ± 0.05	230.13 ± 8.27	5.90 ± 0.0457	6.26 ± 0.129
VOL060S	7.39 ± 0.08	2.93 ± 0.07	113.4 ± 3.22		5.74 ± 0.236

The printlets were found to be gradually opaquer as the relative amount of warfarin sodium in the formulation increased, with VOL005L and VOL005S printlets appearing transparent while turbidity was observable from VOL020 printlets onwards (Fig. 1). This could be due to water loss during the post-curing process, which resulted in the precipitation of warfarin sodium in printlets with higher drug loading.

**Fig. 2:**
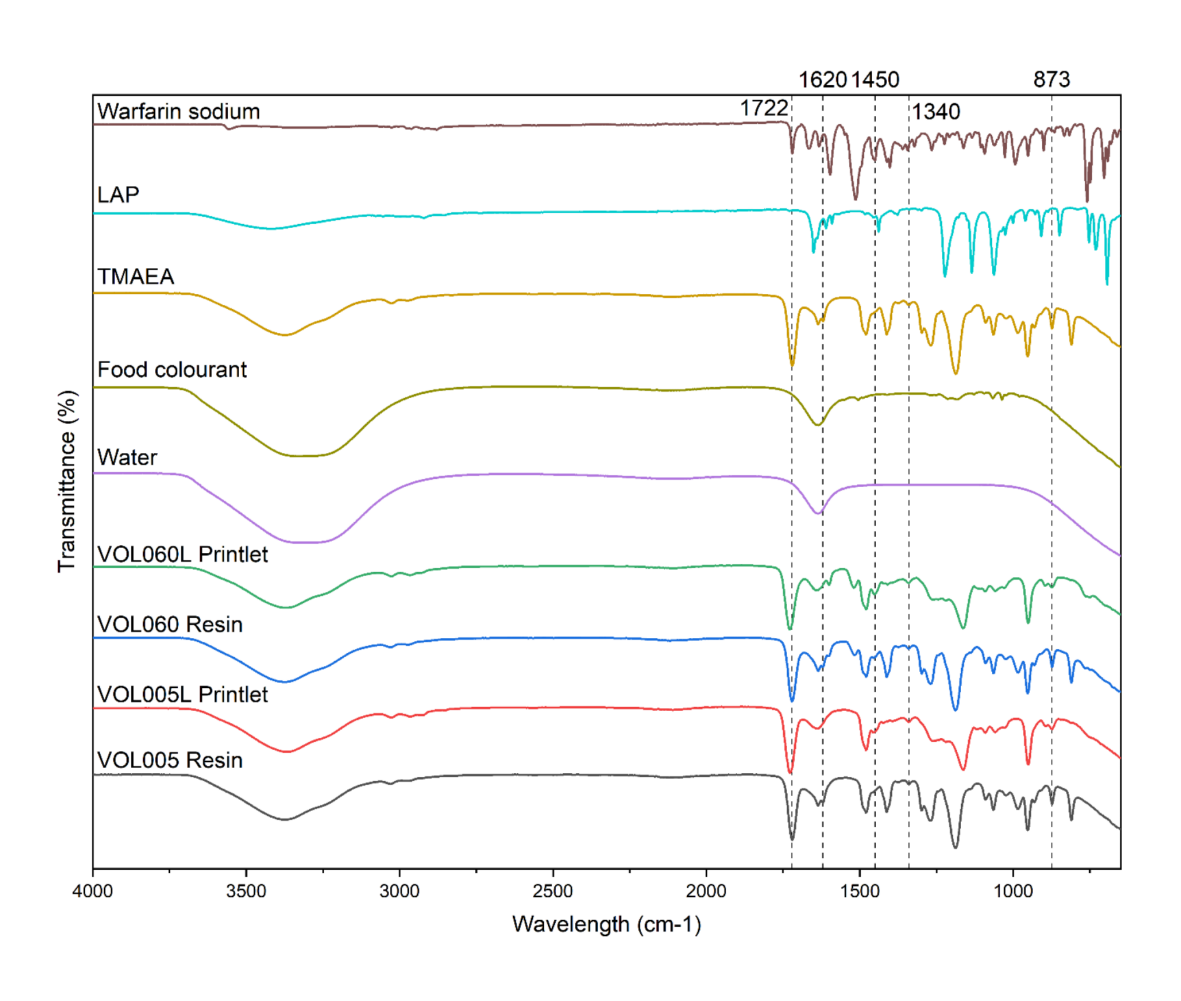
FTIR spectra of warfarin sodium, LAP, TMAEA, food colourant, water, and VOL060 and VOL005 resin and printlets. Absorption bands at 1722 cm^− 1^ (ester C = O stretch), 1620 cm^− 1^ (alkene C = C stretch), and 873 cm^− 1^ (alkene C = C bend) were assigned to the acrylate groups in TMAEA. The signal at 1450 cm^− 1^, corresponding to alkane C-H bend, represents the alkane groups formed following radical polymerization of the acrylate groups. Vibrational peaks at 1340 cm^− 1^ (phenol O-H bend) are assigned to the phenol group in warfarin sodium

FTIR spectroscopy was used to confirm that radical polymerisation between TMAEA monomers had occurred, as well as the incorporation of warfarin sodium into the matrix (Fig. 2). Photopolymerisation was confirmed by analysing the absorption bands assigned to the acrylate groups in TMAEA, namely 1722 cm^− 1^ (ester C = O stretch), 1620 cm^− 1^ (alkene C = C stretch), and 873 cm^− 1^ (alkene C = C bend) [36]. Attenuation of the peak at 1620 cm^− 1^ and 873 cm^− 1^ in the printlet FTIR spectra compared to the resin FTIR spectra is indicative of acrylate radical polymerisation, as C = C bonds are broken and converted to C-C bonds during the reaction. An increase in the signal at 1450 cm^− 1^, corresponding to alkane C-H bend, in the printlet FTIR spectra compared to the resin FTIR spectra further supports the conversion of acrylate groups to alkane groups as a result of the radical polymerisation reaction [49]. The spectrum obtained from warfarin sodium powder showed characteristic vibrational peaks at 1722 cm^− 1^ (lactone C = O stretch) and 1340 cm^− 1^ (phenol O-H bend). However, these peaks were largely masked by stronger signals arising from TMAEA, given the significantly higher proportion of TMAEA present in the printlet and resin samples compared to warfarin sodium.

**Fig. 3:**
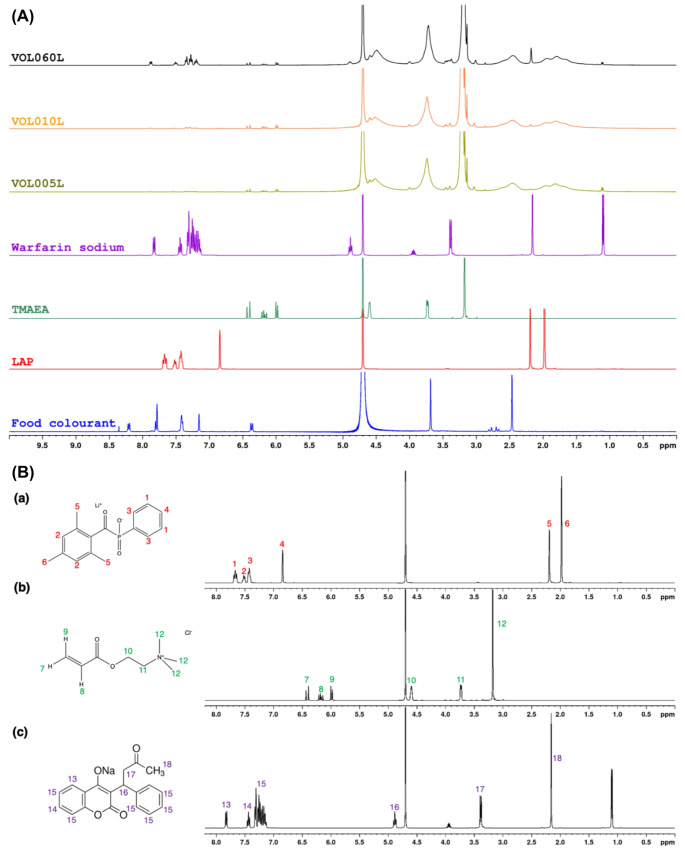
(**A**) ^1^H NMR spectra (D_2_O) of TMAEA, LAP, warfarin sodium, food colourant, and VOL005L and VOL060L samples. (**B**) ^1^H NMR spectra (D_2_O) of (a) LAP, (b) TMAEA, and (c) warfarin sodium clathrate. NMR signals have been assigned to relevant protons in the chemical structure of LAP, TMAEA, and warfarin sodium clathrate

Consequently, to confirm the successful incorporation of warfarin sodium into the printlet matrix, ^1^H-NMR spectroscopy was performed (Fig. 3A). The assignment of ^1^H signals for TMAEA, LAP, and warfarin sodium are provided in Fig. 3B. The signals from the phenyl protons (signal H-13, H-14, and H-15) and methyl protons adjacent to the carbonyl oxygen (H-18) of warfarin sodium, occurring at 7.10–7.80 ppm and at 2.17 ppm respectively, remain visible in the spectra of VOL060L and VOL010L. These signals are only present in trace amounts and barely visible in the spectra of VOL005L, likely because the relative amount of warfarin sodium present in VOL005L falls below or is close to the detection limit of the NMR spectrometer. These confirm the successful integration of warfarin sodium into the polymer matrix with no evidence of any undesired chemical reactions between warfarin sodium and other components of the formulation. The signals occurring at 1.10 ppm and 3.95 ppm on the spectra of warfarin sodium is associated with the proton signals from isopropanol, specifically the CH proton adjacent to the two CH_3_ groups and the CH_3_ protons, respectively. This is observed as warfarin sodium clathrate contains 8.3% isopropyl alcohol [50]. NMR spectroscopy was also used to confirm photopolymerisation had occurred and the incorporation of TMAEA into the polymer matrix. The signals at 6.0–6.5 ppm on the spectra of TMAEA correspond to the CH = CH2 acrylate protons, which are only present in trace amounts in the spectra of VOL005L and VOL060L. During the photopolymerisation reaction, the acrylate C = C double bonds undergo homolytic bond fission to propagate monomer and form the saturated C-C polymer backbone. Therefore, the presence of trace signals arising from the acrylate protons suggest that the photopolymerisation reaction had occurred to near completion. Signals from the methyl groups bonded to the positively charged nitrogen of TMAEA, occurring at 3.2 ppm, remain visible in the spectra of VOL060L and VOL005L. This indicates that TMAEA monomers were successfully integrated into the polymer chain.

**Fig. 4:**
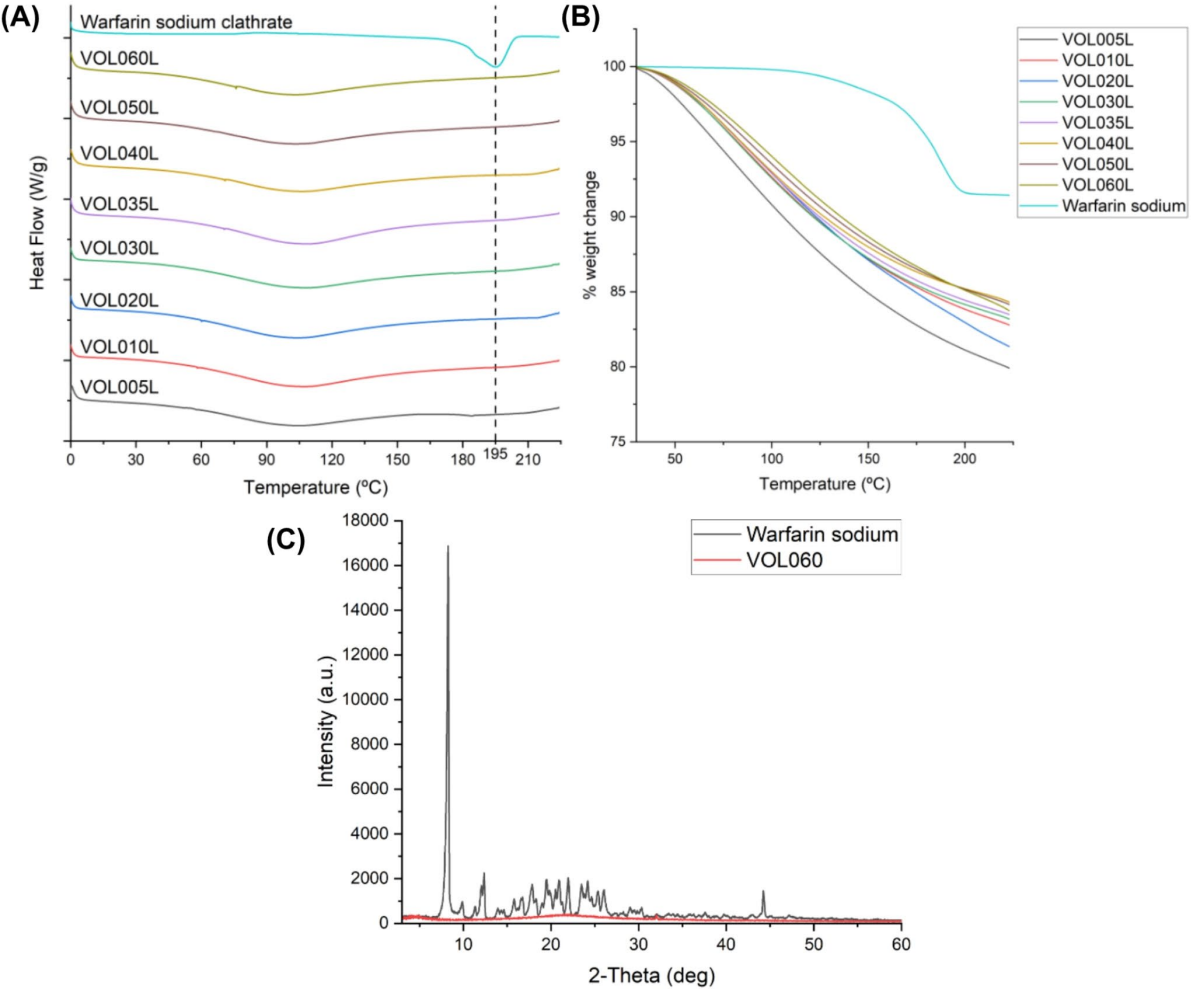
(**A**) DSC thermogram and (**B**) TGA thermogram of warfarin sodium clathrate, and VOL060L, VOL050L, VOL040L, VOL035L, VOL030L, VOL020L, VOL010L, and VOL005L printlets. Endothermic peak at 195 ºC is attributed to the evaporation of isopropyl alcohol and the melting of crystalline warfarin sodium. (**C**) X-ray diffractogram of warfarin sodium clathrate and photopolymerised VOL060 disc. Absence of crystalline peaks characteristic of warfarin sodium in VOL060 suggest that the drug was molecularly dispersed in the formulation

**Fig. 5:**
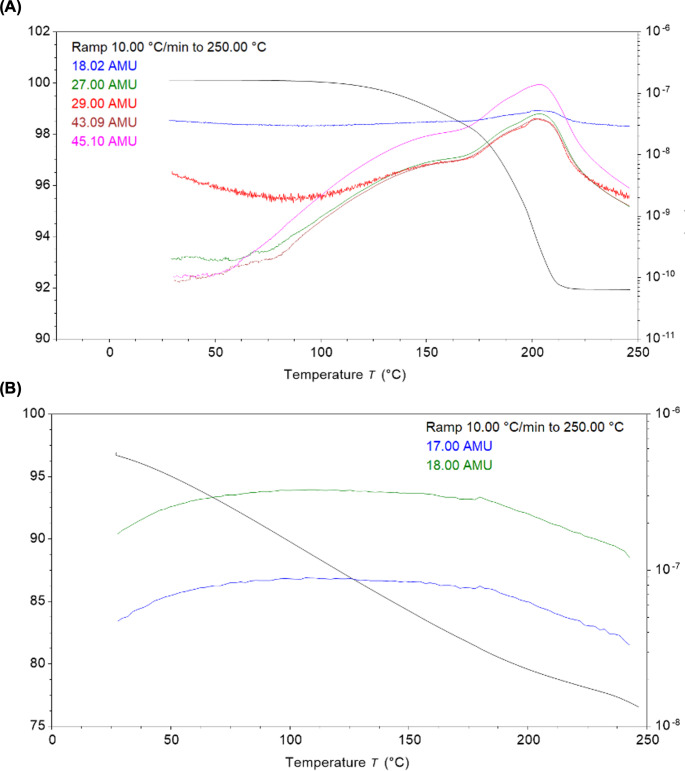
(**A**) TGA-MS of warfarin sodium clathrate, showing mass loss (left y-axis) and ion current (right y-axis (log scale) for ions at m/z 45, 43, 29 and 27 (IPA base peak and principal fragment ions) (**B**) TGA-MS of VOL060L, showing mass loss (left y-axis) and ion current (right y-axis (log scale) for ions at m/z 18 and 17 (water base peak and principal fragment ion)

DSC and XRD was performed to investigate the physical state of warfarin sodium in the printlets. The DSC thermogram of warfarin sodium clathrate shows a distinct endothermic peak at 195 ºC, which is attributed to the evaporation of isopropyl alcohol from the isopropanol clathrate and the melting of crystalline warfarin sodium (Fig. 4A) [51]. This is supported by TGA analysis, wherein a decrease in weight was observed from approximately 170 to 190 ºC (Fig. 4B). TGA-MS performed on the warfarin sodium clathrate sample confirm this mass loss is the evolution of IPA, with an increase in ion current for ions at m/z 45, 43, 29 and 27 amu (base peak and principal fragment ions for IPA) tracking the mass loss. Though there is a very small increase in the ion current for m/z 18 amu (base peak for water) the identical shape of the evolution leads us to conclude that it is from a minor fragment ion associated to IPA and is not a result of water loss i.e. we do not believe any water is lost from the warfarin sodium clathrate (Fig. 5A). The endothermic melting peak of warfarin sodium is absent in the thermograms of all the printlets. Instead, a broad endotherm occurring from 60 to 150 ºC is observed in the printlets’ thermograms, which corresponds to the evolution of water. This is again supported by TGA analysis, which shows a gradual loss of weight from 30 to 225 ºC (Fig. 4B). TGA-MS performed on sample VOL060L confirms this is a continuous slow evolution of water, with an increase in ion current for m/z 18 and 17. No evolution of IPA is evident (Fig. 5B) – this suggests that IPA molecules that were initially bound in the warfarin sodium clathrate lattice were released as free IPA upon dissolution during resin preparation, and subsequently evolved during the post-curing process.

The absence of the melting endotherm of crystalline warfarin sodium in the printlets’ thermograms may suggest that warfarin sodium remained molecularly dispersed in the solid printlets. However, it is also likely that the amount of crystalline warfarin sodium in the printlet samples were below the detection limit of the calorimeter. XRD analysis of warfarin sodium clathrate powder and printlet samples gave similar observations. The acquired X-ray diffractograms of photopolymerised VOL060 XRD disc showed no crystalline peaks that were observed in the X-ray diffractogram of pure warfarin sodium clathrate powder (Fig. 4C). As per DSC analysis, while this may suggest that warfarin sodium was molecularly dispersed in the printlets, the amount of crystalline warfarin sodium could also be below the XRD detection limit.

**Fig. 6:**
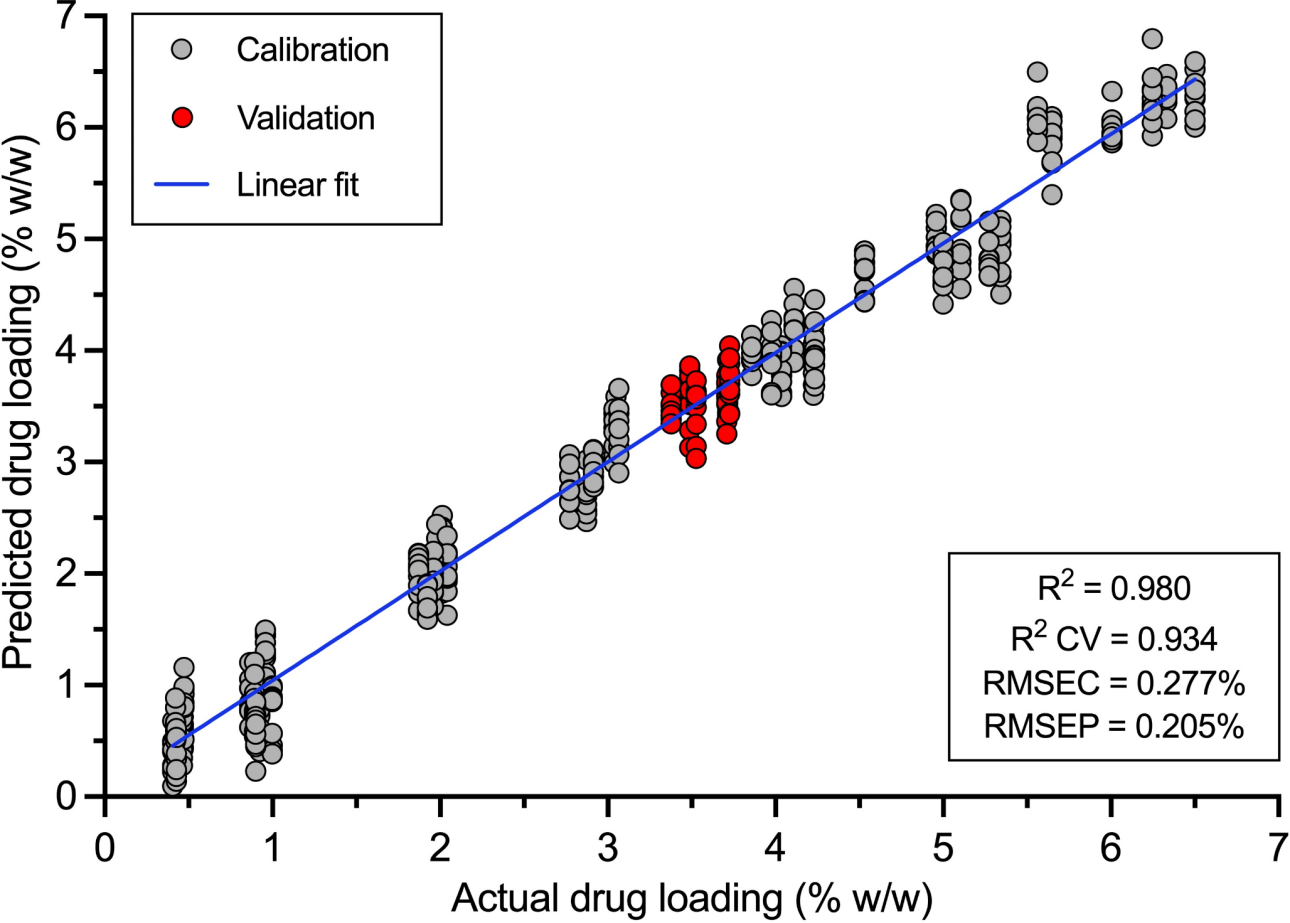
PLSR model for predicting warfarin sodium loading in printlets through NIRS with correlation to HPLC drug loading results. Grey circles represent the samples used to calibrate the model, while red circles represent samples used to validate the accuracy of the model

8 out of the 9 formulations were used to build the calibration model for quantifying the warfarin sodium loading in the printlets (Fig. 6) where the VOL035 formulation printlets were used for model validation. VOL035 was selected as the formulation for model validation as it is close to the 50th percentile of warfarin sodium concentration across formulations, whilst allowing the calibration points to be spaced at equal intervals. The PLSR model with 8 latent variables was developed using following subsets of the acquired spectra: 926–945, 1025–1038, 1118–1162, 1180–1273, 1316–1354, 1385–1434, 1477–1533, 1564–1630, 1645–1675 nm. The model showed strong linearity, with a coefficient of determination of 0.980 and 0.906 based on 10-fold cross-validation. Moreover, the calibration error (root mean square error of calibration (RMSEC)) was only 0.277%, whilst the prediction error (root mean square error of prediction (RMSEP)) was as low as 0.205% in the 3.5% w/w warfarin sodium validation printlets. Therefore, NIRS provided an accurate method for dose verification of warfarin sodium loading in vat photopolymerisation 3D printed matrices. A previous study has reported on the development of an accurate NIRS quantitative model for determining the warfarin dose in a mix of commercial and laboratory compressed pharmaceutical tablets [52]. On the other hand, for vat photopolymerisation, NIRS has mainly been adopted for monitoring of polymerisation reaction parameters such as crosslinking and curing, and no study to date has reported on NIRS chemometric models for accurately quantifying pharmaceuticals in vat photopolymerised 3D printed tablets [53].

**Fig. 7:**
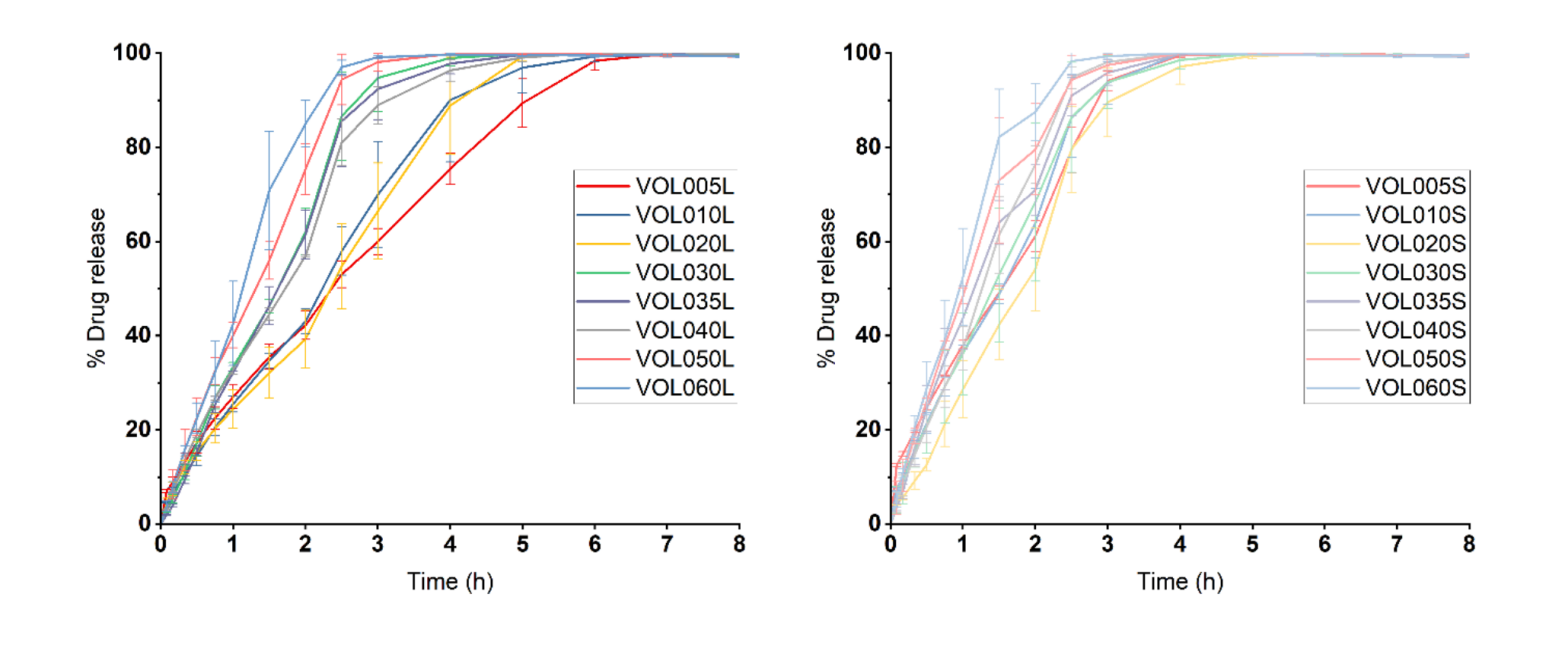
In vitro drug release data of large printlets (left) and small printlets (right) (*n* = 3)

In vitro drug release studies found that complete solubilisation and drug release was achieved in 2.5–7 h, depending on the drug load and size of the printlet (Fig. 7). The drug release rates from any of the small or large 3DP matrices were not influenced by the pH of the dissolution medium, indicating a time-dependant drug release and that inter-patient differences in gastrointestinal pH conditions would not negatively impact the drug release. The achieved drug release rates are significantly faster than most previous vat photopolymerised pharmaceutical dosage forms. Notably, in our previous study that reported DLP printed warfarin sodium printlets wherein poly(ethylene glycol)-diacrylate (PEGDA) was used as the matrix monomer, slightly less than 80% drug release was achieved after 24 h [21]. Generally, drug release rate increased as the amount of warfarin sodium loaded in the printlets increased. For example, VOL005L reached 100% drug release after 7 h while it only took VOL060L 3 h to do so. This could likely be due to inter-polymer supramolecular interactions being in competition with polymer-drug interactions. As the relative amount of warfarin sodium increases, the extent of supramolecular interactions between poly-TMAEA chains decreases as more warfarin sodium molecules are available to form similar interactions with the polymer chains. Consequently, the overall strength of inter-polymer supramolecular interactions in the printlet decreases as the drug load increases, resulting in faster printlet solubilisation and drug release rates [54].

**Fig. 8:**
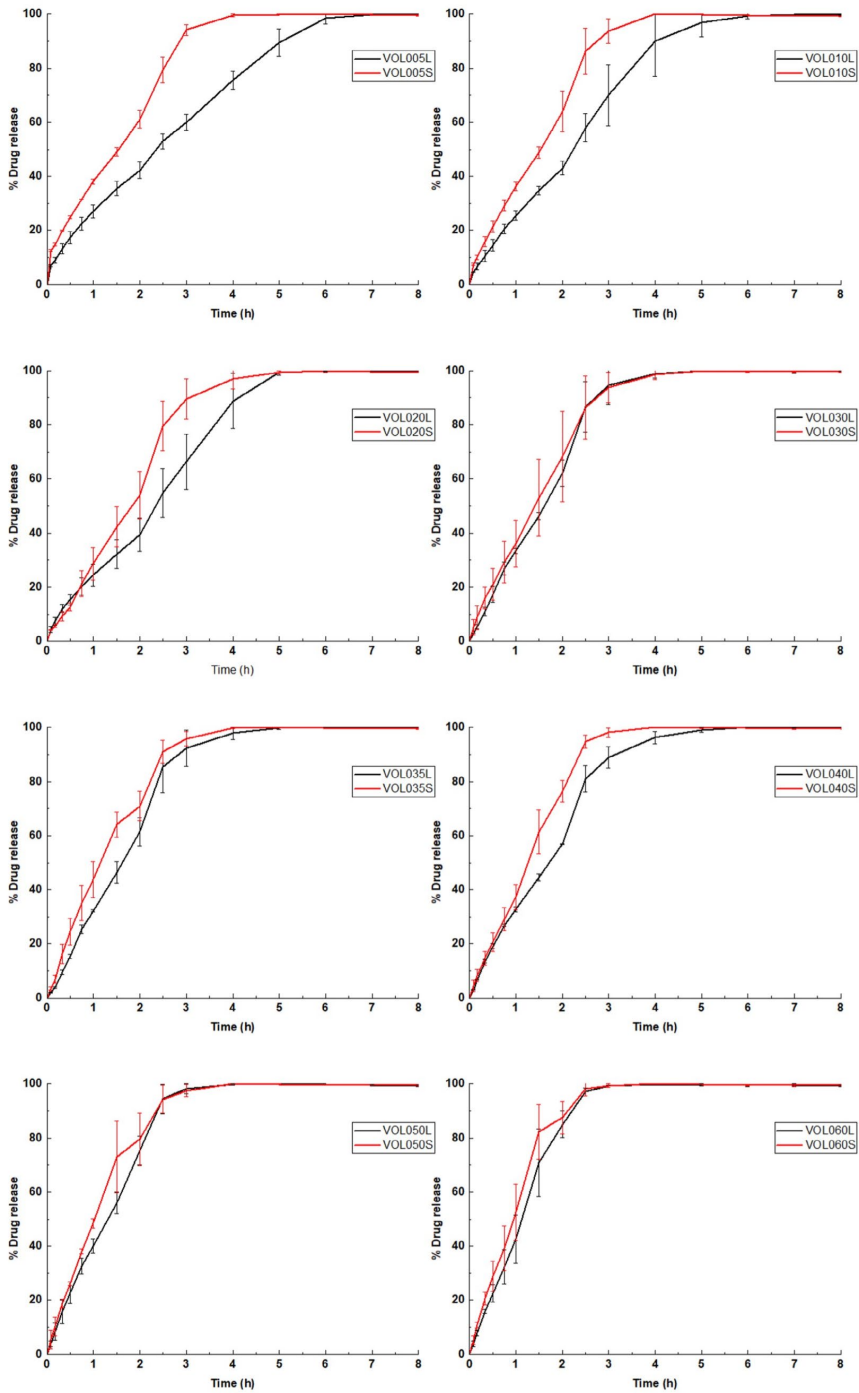
Comparison of drug release profiles of small and large printlets derived from the same formulations

Drug release rates were also faster in small printlets compared to their large equivalents, up to 2% w/w warfarin loading (VOL020) (Fig. 8). This is because of the higher surface area to volume ratio that the small printlets affords compared to large printlets, resulting to accelerated drug release rates in accordance with the Noyes Whitney equation. However, drug release rates were almost equivalent between small and large tablets with warfarin sodium loads above 2% w/w. This could be because drug release rates were being limited, and hence largely governed, by the solubilisation of warfarin sodium, given the hypothesised precipitation of warfarin sodium in printlets with higher drug loading. A potential reversion in solid state of warfarin sodium clathrate to the non-ionised acidic warfarin form may negatively impact the dissolution rate of the compound; however, as faster dissolution rates in the printlets with higher drug loading, that were visually opaquer, were observed, this scenario was most likely not the case for the developed printlets [55].

As such, the findings from the in vitro drug release studies revealed both complete matrix and drug solubilisation within 7 h and suggest that the drug release profiles may be personalised according to the patient’s therapeutic needs by altering the drug dose and/or size of the printlet. A recent study reported the DLP 3DP of printlets containing TMAEA, PEGDA, and acetylsalicylic acid (ASA) as model drug, also a freely water-soluble drug like warfarin sodium clathrate [56]. Due to the inclusion of PEGDA, the 3DP matrices exhibited greater swelling and prolonged drug release rates at frequent media changes, as compared to the developed formulations in our work. Moreover, as crosslinked PEGDA matrices are insoluble in water and exhibit long biodegradation times, a gastric obstruction risk may be encountered if administered orally to patients [57]. Additionally, studies thus far reporting the production of warfarin sodium-loaded tablets using vat photopolymerization have been limited by their insolubility in water owing to reliance on cross-linking monomers such as PEGDA [21]. Therefore, the study herein represents an improvement in the production of warfarin sodium-loaded printlets via vat photopolymerization through the development of a formulation that produces water-soluble drug-loaded hydrogels. It is conceivable that water-soluble printlets loaded with other drugs, especially those readily soluble in TMAEA, may also be produced with the same or similar formulations; future studies may evaluate the diversity of drugs for which the reported formulation is amenable to. In addition, inter-patient differences in gastrointestinal pH conditions would not negatively influence the drug release from the printlets which were seen to be independent of media pH.

The present study demonstrates the feasibility of producing water-soluble drug-loaded hydrogel with volumetric printing. Whilst the significantly accelerated printing speeds support the use of the technology in fast-paced clinical settings, there remain limitations that must be addressed. Specifically, the high feedstock-to-print ratio (or amount of wasted feedstock per print) is a drawback of volumetric printing when compared to other types of vat photopolymerization technologies. Previous work by our research group has demonstrated the feasibility of printing multiple objects in the same cuvette, and therefore reducing the amount of wasted feedstock discarded per print [33]. The number of prints per print cycle is theoretically limited by the size of the cuvette and light projection. Nonetheless, due to the inability to reuse excess resin, the high volume of waste persists. Therefore, strategies to recycle resins in a high throughput and chemistry-agnostic manner would enhance the usability of volumetric printing.

## Conclusion

Volumetric 3D printing was successfully used to fabricate warfarin-loaded printlets with varying amounts of warfarin sodium within 6.5–11.1 s. All printlets were found to solubilise completely in the in vitro dissolution media within 2.5–7 h, and their release profiles were shown to be tuneable by changing the relative loading of warfarin sodium and the size of the printlet. The PLSR model developed from the printlets’ NIR spectra showed strong linearity and high prediction accuracy in non-destructively quantifying the amount of warfarin sodium loaded in the printlets. As such, this study demonstrates a high throughput method of producing water-soluble, personalised printlets via vat photopolymerisation and accurately confirming the amount of drug incorporated into these printlets. Through further validation of the NIR model’s robustness and extensive optimisation of printing parameters to achieve even more consistent prints, volumetric 3D printing may eventually become a powerful technology for fabricating personalised medicines that is suited to meet the demands of fast-paced clinical environments.

## Data Availability

The data supporting the reported findings are available from the corresponding author upon request.
